# Supplementary material for: Causal interplay between lactose intolerance and gut microbiota: a combined bidirectional Mendelian randomization and in vivo validation study
Source: Front Nutr. 2026 Jun 1;13:1803337. doi: 10.3389/fnut.2026.1803337 (PMC13265576; doi:10.3389/fnut.2026.1803337)
Supplement: Supplementary file 1 [file Data_Sheet_1.zip › supplementary materials/Forward/forest plot/ebi-a-GCST90027749.finngen_R12_E4_LACTONAS.pdf]

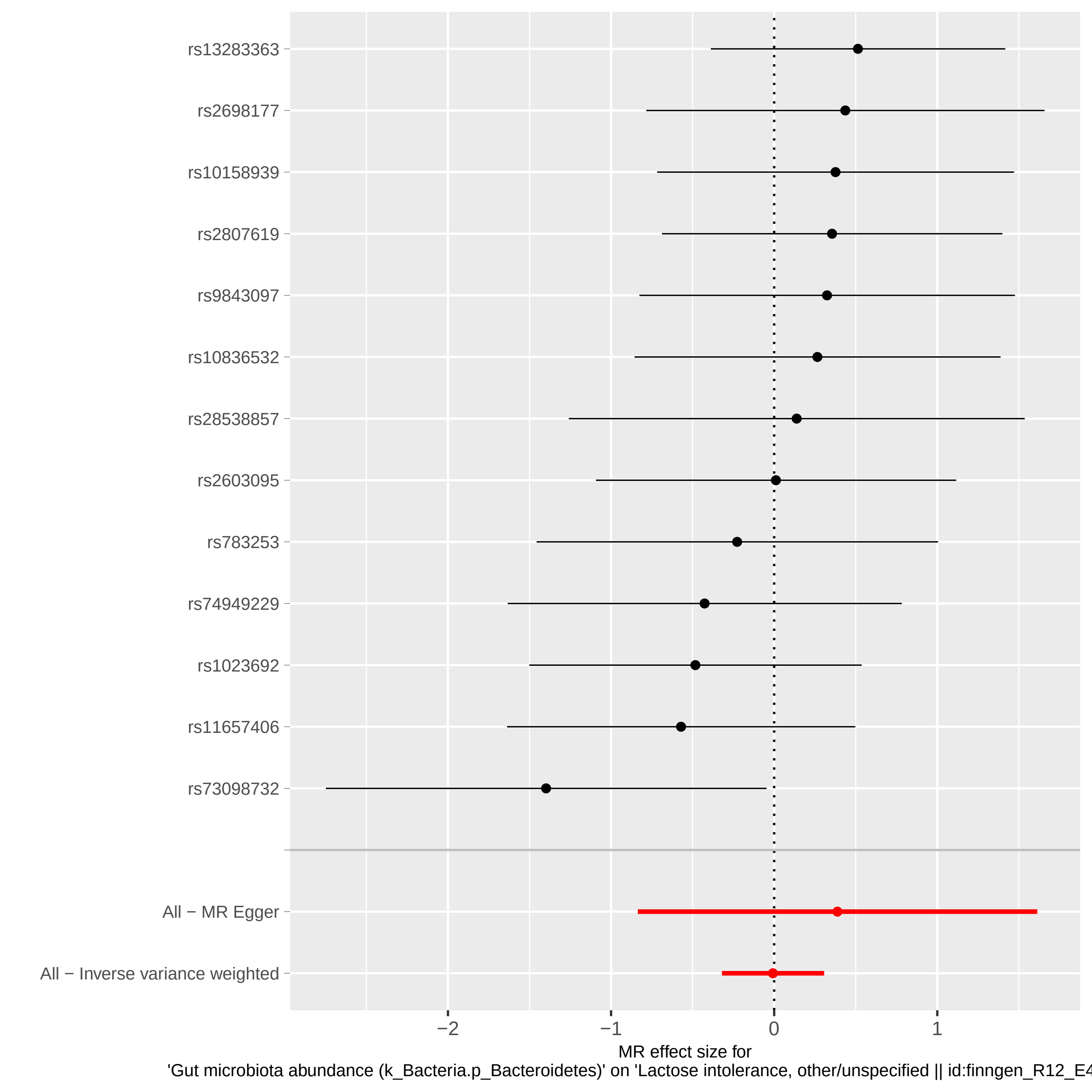

rs13283363
rs2698177
rs10158939
rs2807619
rs9843097
rs10836532
rs28538857
rs2603095
rs783253
rs74949229
rs1023692
rs11657406
rs73098732
All − MR Egger
All − Inverse variance weighted
−2
−1
0
1
MR effect size for
'Gut microbiota abundance (k_Bacteria.p_Bacteroidetes)' on 'Lactose intolerance, other/unspecified || id:finngen_R12_E4
